# Comparison of heroin and fentanyl use in US nationally representative surveys

**DOI:** 10.1186/s13722-025-00539-0

**Published:** 2025-02-11

**Authors:** Jarratt D. Pytell, Paul J. Christine, Katherine LeMasters, Karilynn M. Rockhill, Joshua C. Black, Richard C. Dart, Ingrid A. Binswanger

**Affiliations:** 1 Division of General Internal Medicine, University of Colorado School of Medicine, Aurora, CO USA; 2 Department of General Internal Medicine, Denver Health Hospital Authority, Denver, CO USA; 3 Institute for Health Research, Kaiser Permanente Colorado, Aurora, CO USA; 4 Department of Epidemiology, School of Public Health, University of Colorado Medical Campus, Aurora, Colorado USA; 5 Rocky Mountain Poison and Drug Safety, Denver Health and Hospital Authority, Denver, Colorado USA; 6 Department of Emergency Medicine, University of Colorado School of Medicine, Aurora, CO USA; 7 Chemical Dependency Treatment Services, Colorado Permanente Medical Group, Denver, CO USA; 8 Department of Health Systems Science, Kaiser Permanente Bernard J. Tyson School of Medicine, Pasadena, CA USA; 9 Department of Medicine, University of Colorado School of Medicine, Mail Stop B180, 12631 E. 17th Ave, 80045 Aurora, CO USA

## Abstract

**Background:**

Given the opioid overdose crisis, surveillance of evolving opioid use patterns is critical to the effective deployment of mitigation strategies. The National Survey on Drug Use and Health (NSDUH) provided the first annual US estimate of illicitly manufactured fentanyl (IMF) use in 2022. However, as a household survey, NSDUH may not capture the full extent of population heroin and IMF use. We compare estimates of past-year heroin and IMF use and correlates of use in NSDUH and the Survey of Non-Medical Use of Prescription Drugs (NMURx) survey which employ an alternate sampling strategy.

**Methods:**

We conducted a cross-sectional analysis of the 2022 NSDUH and NMURx. NSDUH samples respondents using a probability-based approach targeting community-dwelling individuals, while NMURx samples respondents using an opt-in, online survey panel. US adults ages 18 years and older were included. The main outcomes were differences in the weighted percentage of population reporting past-year use of heroin, IMF, and either heroin or IMF between the surveys. Secondary outcomes were the patterns of association of past-year heroin or IMF use with comorbid substance use, treatment utilization, and demographic characteristics between the surveys. Data were analyzed March to June 2024.

**Results:**

NSDUH (*n* = 47,100 respondents) had a lower proportion of respondents who identified as non-Hispanic White and graduated college, and a higher proportion with past week employment than NMURx (*N* = 59,041 respondents). Past-year use of heroin, IMF, and either heroin or IMF were lower in the NSDUH than the NMURx. NSDUH estimated 0.52% (95% CI: 0.40%, 0.69%) %) of the US population used either heroin or IMF in the past year compared to 1.05% (95% CI: 0.97%, 1.14 0) in NMURx. In regression models, stimulant and benzodiazepine use were consistently associated with increased heroin or IMF use across both surveys.

**Conclusions and relevance:**

The estimated prevalence of heroin or IMF use was nearly 50% higher in the NMURx compared to NSDUH. These results highlight the importance of using complementary surveillance approaches to obtain accurate estimates of the prevalence and patterns of heroin or IMF use.

**Supplementary Information:**

The online version contains supplementary material available at 10.1186/s13722-025-00539-0.

## Introduction

Addressing the national overdose crisis requires access to timely and accurate data on the prevalence of drug use and the characteristics of people who use drugs in the general population [1, 2]. Clinicians, public health researchers, policymakers, community organizations, and advocates rely on data to tailor interventions and identify emerging trends in the drug supply and communities most impacted by the overdose crisis. The National Survey on Drug Use and Health (NSDUH) is a household survey that has been conducted since 1971 and provides detailed information about substance use in the United States (US) on an annual basis [3]. As with all surveys, the NSDUH has limitations, including delays in updating survey questions to reflect changes in the drug supply, time lags between data collection and public availability, and the exclusion of unhoused individuals which potentially lead to underestimates of substance use in the US.

The US overdose crisis after 2017 is attributed to the rise of illicitly manufactured fentanyl (IMF), a high-potency synthetic opioid, in the illicit opioid supply [4]. The NSDUH included questions about IMF for the first time in 2022 and found that 0.2% of the US population aged 12 and older used IMF in the past year, while 0.4% used heroin [5, 6]. The relatively higher rates of heroin use compared to IMF use found in NSDUH does not correspond with other data indicating a decline in heroin use and a rise in IMF use in the US [7]. For example in 2023, of the 78,226 opioid overdose-related deaths, 92% (*n* = 71,821) involved synthetic opioids like fentanyl (excluding methadone), whereas only 5% (*n* = 3,929) involved heroin [4]. Beyond mortality, a national toxicology laboratory reported that in 2021 nearly 6% of urine toxicology tests were positive for fentanyl, compared to less than 1% for heroin [8]. The discrepancy between NSDUH estimates and other public health data underscores the importance of complementing NSDUH data with other national datasets to better capture the evolving landscape of opioid use.

To enhance our understanding of opioid use in the US, this study compares NSDUH findings on heroin and IMF use with those from the Survey of Non-Medical Use of Prescription Drugs (NMURx), another US nationally representative survey, which assesses non-medical use of prescription drugs and substance use in the US. We sought to highlight how differences in survey design influence heroin and IMF use prevalence estimates and patterns of association. To accomplish this aim, we first compared national estimates of heroin and IMF use between the two surveys. Next, we measured and compared patterns of association between past-year heroin or IMF use and relevant demographic, substance use, and treatment factors in both surveys.

## Methods

### Data sources

The Research Abuse, Diversion, and Addiction-Related Surveillance (RADARS) System developed and validated the NMURx, which was externally validated through alignment with national benchmarks from probability-based surveys, including the NSDUH [9]. Briefly, NMURx uses a cross-sectional, nonprobability design where respondents complete a self-administered, online questionnaire. NMURx participants opt-in to a commercial survey firm and are recruited to participate in various surveys to receive remuneration. Data are collected biannually, with survey weights producing national estimates for ages ≥ 18 years. Respondents must have access to the internet through a computer or smartphone device. There are no exclusion criteria based on housing status. Internal data consistency checks identify and removes completed questionnaires exhibiting improbable response patterns. Data from the 2022 NMURx survey waves were adjusted to represent the US general public ages ≥ 18. NMURx respondents are asked about heroin, IMF, and fentanyl analog use, with questions randomized to reduce order bias (Table 1) [10]. 

**Table 1:** Comparison the NMURx and NSDUH Survey characteristics in 2022

Survey	Design and Methods	Heroin Ascertainment	Illicitly Manufactured Fentanyl Ascertainment
Survey of Non-Medical Use of Prescription Drugs (NMURx)	• Nonprobability sample. • Weighted to represent adults ages ≥ 18 years • Participants seek opportunity to complete surveys for payment from a commercial survey firm. • Focus on nonmedical use of prescription drugs and substances • Online only • Random order of substance use items	Have you ever used heroin (Brown sugar, Cheese, China White, Dope, H, Horse, Junk, Skag, Skunk, Smack, White Horse)?	Have you ever used fentanyl not made by a drug company (Apache, China Girl, China White, Dance Fever, Friend, Goodfella, Jackpot, Murder 8, Tango and Cash, TNT)? Have you ever used drugs similar to fentanyl (carfentanil, acetylfentanyl, or furanylfentanyl)?
National Survey on Drug Use and Health (NSDUH)	• Probability sample • Weighted to presented non-institutionalized adults ages ≥ 12 years • Participants recruited based on household sampling by a federal contractor. • Focus on substance use, mental health, and treatment. • In-person or online • Static order of substance use items o There are 49 modules. Heroin is asked in module 9 while IMF is asked in module 39.	These next questions are about heroin. Have you ever, even once, used heroin?	This next question is about illegally made fentanyl, which is fentanyl that people can’t get from a doctor or pharmacy. Illegally made fentanyl can come in forms such as powder, pills, or blotter paper. It can also be mixed with heroin or other drugs. Have you ever, even once, used illegally made fentanyl?

NSDUH provides annual estimates of tobacco, alcohol, and drug use in a cross-sectional, nationally representative household survey of the non-institutionalized US public aged ≥ 12 years. A detailed description of NSDUH survey methodology is available elsewhere [3]. Starting in 2021, NSDUH used multimodal data collection and, in 2022, 42% of interviews were completed online and 58% completed in-person using audio-computer assisted self-interview to reduce desirability bias [5]. The NSDUH survey instrument includes questions about the use of all classes of prescribed and nonprescribed drugs. The order of the questions is the same for all respondents. NSDUH has included questions about heroin use since its inception in 1971 [11] and added questions about the use of IMF in 2022 (Table 1).

### Heroin use, IMF use, and either heroin or IMF use

We identified respondents with past-year use of heroin or IMF and further classified the recency of use as past-month or past 2–12 months. NMURx and NSDUH definitions of past-month use slightly differed. NMURx reported past-month as use in the last “4 weeks” while NSDUH used “past 30 days.” Respondents might be unaware if IMF is in their heroin supply or consider “heroin” as general term for nonprescription opioids. Therefore, we constructed a third variable to capture most recent use of either heroin or IMF to provide an overall assessment of the population who is likely exposed to IMF.

### Respondents characteristics

Covariates were chosen to describe the demographic, socioeconomic, comorbid substance use, and treatment factors related to opioid use. We were limited to covariates that have similar questions and response scales in both NSDUH and NMURx (Supplement eTable 1). Demographic and socioeconomic factors included age, sex, race and ethnicity, income, highest level of education, marital status, past week employment, and health insurance status. Comorbid substance use factors included frequency of cigarette use, past-year cannabis use, past-year illicit stimulant use (cocaine or methamphetamine), and past-year nonmedical benzodiazepine use. Respondents were classified having heavy alcohol use according to the National Institutes of Alcohol Abuse and Alcoholism definition (≥ 15 drinks per week for men; ≥ 8 drinks per week for women) [12]. Past-year receipt of any medication for opioid use disorder was ascertained by the NSDUH question asking about the receipt of “prescription medication you may have used to cut back or stop your drug use” and a description of methadone, buprenorphine, and naltrexone was provided. In NMURx, past year receipt of medication for opioid use disorder was ascertained by questions asking about the receipt of “prescribed medications for opioid dependence” with an option to select from a list of medications including methadone, buprenorphine, and “other.” For NMURx, the “other” category likely represents adjuvant medications to treat opioid withdrawal and naltrexone. Naltrexone is rarely used to treat opioid use disorder [13] and the adjuvant medications are not considered treatments for opioid use disorder and therefore were not included. Additional covariates include self-reported health status (e.g., Fair/Poor, Good, Very Good, or Excellent) and if the respondent had spent at least one night in the hospital in the past year.

### Analysis

All analyses accounted for the complex survey designs and include the design variables. We first described the sociodemographic, comorbid drug use, and treatment characteristics of NSDUH and NMURx respondents and the recency of past-year use of heroin, IMF, and either heroin or IMF by calculating weighted percentages and 95% confidence intervals (CI).

Given the methodological differences of the surveys, we applied a bootstrap approach to estimate differences and confidence intervals between the two surveys [14]. This approach was necessary because the fundamentally different designs of the NSDUH (probability-based sampling) and NMURx (opt-in, non-probability-based sampling) precluded a direct combination of the surveys for model-based comparisons.

To calculate the differences between the weighted percentages of NSDUH and NMURx respondent characteristics, we took 2000 samples with replacement from the surveys. The mean of the 2000 differences, calculated as the absolute differences between point estimates, gives the estimated difference in point estimates, with percentile-based CIs from the bootstrap distribution [15]. To adjust for multiple testing, we applied a Bonferroni correction to the α level of 0.05 and report the corrected confidence interval (cCI) [16]. 

We modeled the association of past-year heroin or IMF use with respondent characteristics using logistic regression, incorporating clustering variables and analytic weights to account for complex survey designs. We assessed for evidence of multicollinearity and found none using a variance inflation factor cutoff of 5. Since our goal was to describe patterns of association, we presented the adjusted Odds Ratios [aOR] obtained from the logistic regression models and included adjusted average marginal effects from the models in the supplementary material (supplement eFigure 1) [17]. Due to sparse data, the race and ethnicity categories for Native American, Native Hawaiian, Asian, Multiple races, and “Other” were combined into a single group of another non-Hispanic race for the regression modeling. Respondents who left cigarette use blank were included in the “not at all” group for modeling. All analyses were conducted in R version 4.4, used the `survey` and `margins` packages [18, 19]. The RADARS System provided a limited dataset of NMURx and the NSDUH public-access data was used for the analysis [3, 9]. Informed consent had been obtained from all respondents participating in the NMURx and NSDUH surveys, as per the ethical guidelines established by the respective survey protocols. The Colorado Multiple Institutional Review Board determined this study non-human subjects research. Ethics, Consent to Participate, and Consent to Publish declarations: not applicable. Study reporting followed the Strengthening the Reporting of Observational Studies in Epidemiology (STROBE) reporting guideline [20]. 

## Results

### NSDUH and NMURx populations

The 2022 NSDUH survey had 47,100 respondents, and the 2022 NMURx survey had 59,041 respondents across two waves (wave 1: 29,637 and wave 2: 29,404). The final weighted samples were 256,281,676 for NSDUH and 259,008,595 for NMURx. Compared to NSDUH, NMURx had a higher percentage of non-Hispanic White participants (absolute difference: 8.43%, 95% cCI: 7.45%, 9.40%) and college graduates (absolute difference: 8.12%, 95% cCI: 7.21%, 9.09%) and a lower percentage of respondents in the highest income group of ≥$75,000 annually (absolute difference: -7.54%, 95% cCI: -8.52%, -6.57%) and having worked in the past week (absolute difference: -9.28%, 95% cCI: -10.32%, − 8.31%). Age and sex distributions were similar between NSDUH and NMURx (supplement eTable 2). Compared to NSDUH, a lower percentage of the NMURx respondents reported cannabis use (absolute difference − 1.56%, cCI − 8.31%, -0.79%,), heavy alcohol use (absolute difference − 2.97%, cCI − 3.45%, -2.47%), and past-year medications for opioid use disorder (absolute difference − 0.40%, cCI − 0.57%, -0.24%); a higher percentage of NMURx respondents reported not smoking cigarettes (absolute difference 3.82%, cCI 3.19%, 4.49%) and nonmedical benzodiazepine use (absolute difference 0.70%, cCI 0.49%, 0.91%).

### Past-year use of heroin, IMF, and either heroin or IMF

**Fig. 1:**
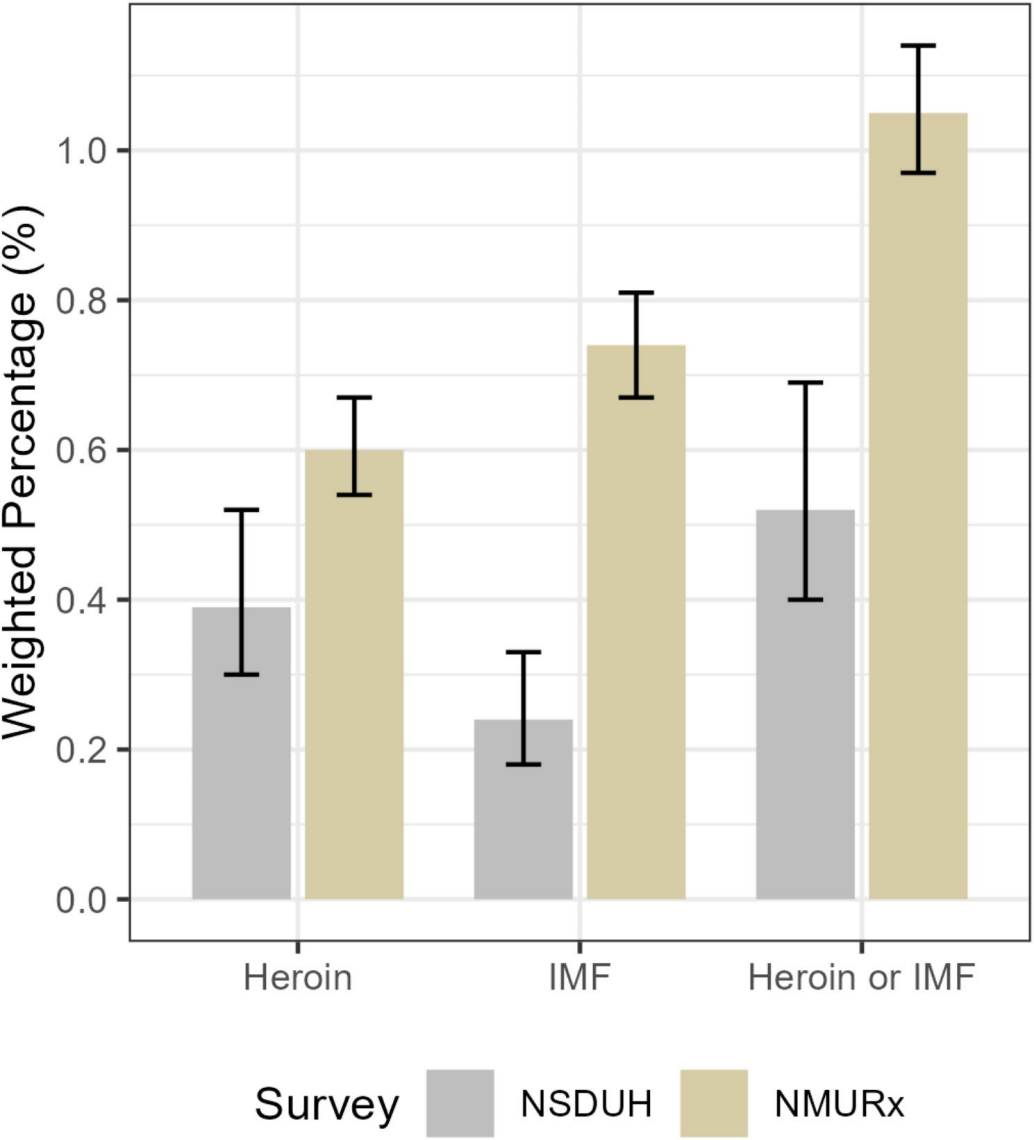
Weighted percentage with past year use of heroin, illicitly manufactured fentanyl (IMF), and either heroin or IMF

Estimates of past-year use of heroin, IMF, and either heroin or IMF were consistently lower in NSDUH compared to NMURx (Fig. 1; Table 2). Past-year heroin use was 0.39% (95% CI: 0.30%, 0.52%) in NSDUH and 0.60% (95% CI: 0.54%, 0.67%) in NMURx, with an absolute difference of 0.21% (cCI: 0.09%, 0.32%). Past-year IMF use was 0.24% (95% CI: 0.18%, 0.33%) in NSDUH and 0.74% (95% CI: 0.67%, 0.81%) in NMURx, with an absolute difference of 0.49% (cCI: 0.39%, 0.60%). Additionally, past-year use of either heroin or IMF was 0.52% (95% CI: 0.40%, 0.69%) in NSDUH and 1.05% (95% CI: 0.97%, 1.14%) in NMURx, with an absolute difference of 0.53% (cCI: 0.38%, 0.67%).

**Table 2:** Weighted percentage of Past Year Heroin, illicitly manufactured fentanyl (IMF), and either heroin or IMF use. **Bonferroni corrected confidence interval. Setting significant level of α = 0.05 and accounting for 43 comparisons, the corrected significant level (α) is equal to 0.05/43 = 0.0011 and we constructed 99.88% confidence intervals. The absolute difference formula: NMURx prevalence minus NSDUH prevalence

**Most recent use**	Heroin			IMF			Heroin or IMF			
	NSDUH *%* *(95% CI)*	NMURx *%* *(95% CI)*	*Abs. Diff.* *(*corrected CI)*	NSDUH *%* *(95% CI)*	NMURx *%* *(95% CI)*	*Abs. Diff.* *(*corrected CI)*	NSDUH *%* *(95% CI)*	NMURx *%* *(95% CI)*	*Abs. Diff. * *(*corrected CI)*	
Past year	0.39 (0.30, 0.52)	0.60 (0.54, 0.67)	0.21 (0.09, 0.32)	0.24 (0.18, 0.33)	0.74 (0.67, 0.81)	0.49 (0.39, 0.60)	0.52 (0.40, 0.69)	1.05 (0.97, 1.14)	0.53 (0.38, 0.67)	
Past Month	0.26 (0.19, 0.38)	0.29 (0.25, 0.33)	0.02 (-0.08, 0.12)	0.10 (0.07, 0.16)	0.41 (0.36, 0.47)	0.31 (0.24, 0.38)	0.32 (0.23, 0.43)	0.57 (0.51, 0.64)	0.25 (0.14, 0.36)	
2–12 months	0.13 (0.09, 0.19)	0.32 (0.27, 0.37)	0.19 (0.12, 0.25)	0.14 (0.08, 0.22)	0.32 (0.28, 0.37)	0.19 (0.11, 0.26)	0.20 (0.14, 0.3)	0.48 (0.42, 0.55)	0.28 (0.18, 0.37)	

### Most recent use of heroin, IMH, and either heroin or IMF

All estimates of the percentage of respondents using heroin, IMF, and heroin or IMF use in the past-month or 2–12 months was lower in NDUH compared to NMURx, with the exception of past-month heroin use which was similar in both surveys (Table 2). The largest estimated difference was in the percentage of respondents reporting past-month IMF use: NSDUH estimated 0.10% (95% CI: 0.07%, 0.16%), whereas NMURx estimated 0.41% (95% CI: 0.36%, 0.47%), an absolute difference of 0.31% (cCI: 0.24%, 0.43%).

### Association of past-year heroin or IMF use with demographic and socioeconomic factors

**Fig. 2:**
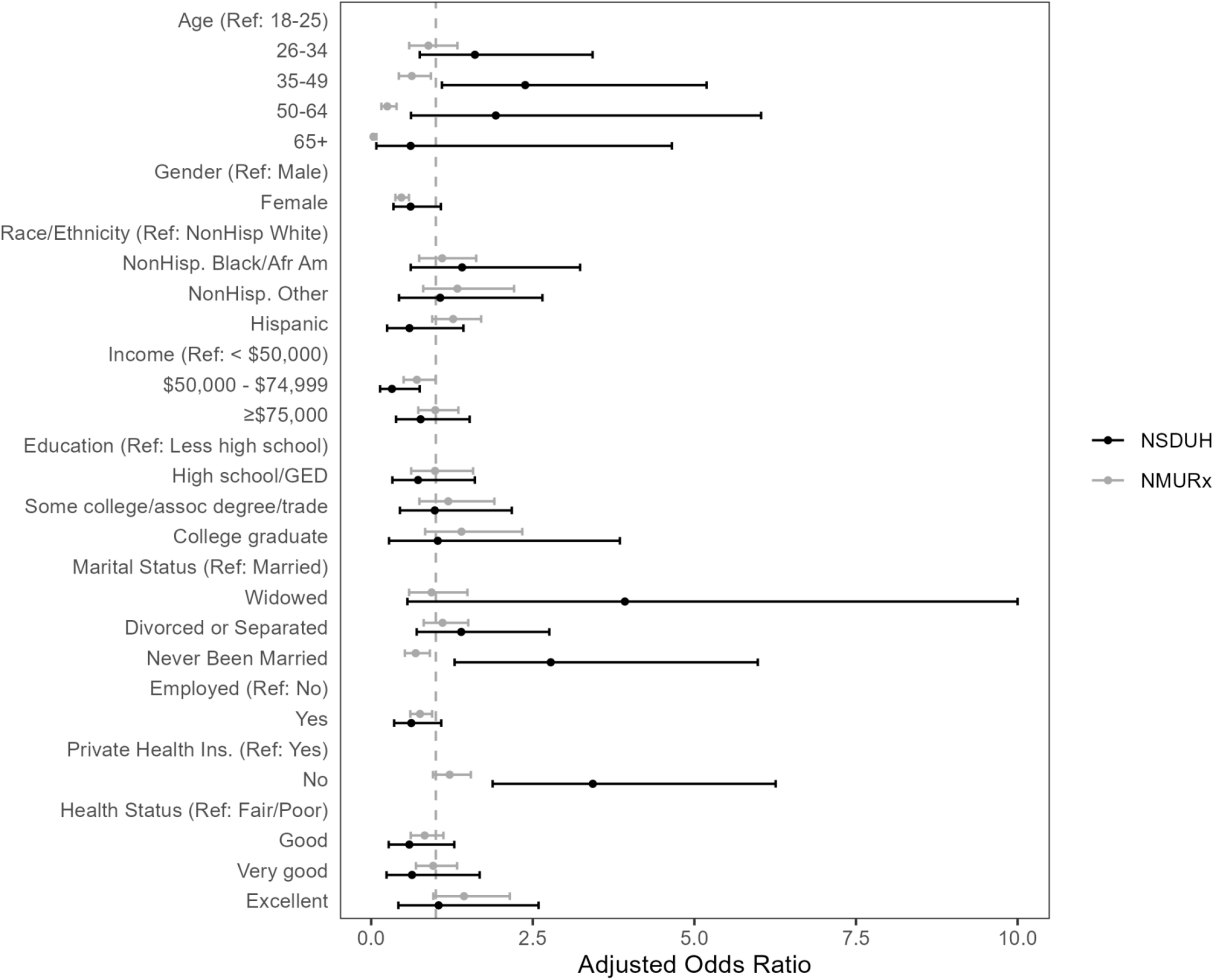
Association of past-year heroin or illicit fentanyl use with demographic, socioeconomic, and self-reported health factors

In fully adjusted logistic regression models, similar patterns in both NSDUH and NMURx were found for income, where respondents in the $50,000-$74,999 group had lower odds of reporting past-year heroin or IMF use (NSDUH: aOR 0.32, 95% CI 0.14, 0.75; NMURx aOR 0.71, 95% CI 0.50, 0.99) relative to <$50,000 (Fig. 2). Several variables showed similar trends across NSDUH and NMURx, although with different levels of statistical precision. For instance, females had lower odds of past-year heroin or IMF in both NSDUH (aOR 0.61, 95% CI 0.34, 1.08) and NMURx (aOR 0.47, 95% CI 0.37, 0.58) compared to males, with NSDUH estimates being less precise and crossing the null value. Past-week employment also showed lower odds of past-year heroin or IMF use compared to those who had no employment, with reduced precision for NSDUH (aOR 0.62, 95% CI 0.36, 1.08) compared to NMURx (aOR 0.76, 95% CI 0.61, 0.94). Additionally, not having private insurance relative to having private insurance had higher odds in NSDUH (aOR 3.43, 95% CI 1.88, 6.26) and a weaker association in NMURx (aOR 1.21, 95% CI 0.96, 1.54) with confidence interval crossing the null value.

Age and marital status had divergent findings between NSDUH and NMURx. In NSDUH, the 35–49 age group had higher odds of past-year heroin or IMF use relative to 18–25 age group (aOR 2.38, 95% CI 1.09, 5.19), with no other differences by age group. In NMURx, the 18–25 age group had the highest odds of past-year heroin or IMF use, with significantly lower odds in other age groups (ages 35–49, aOR 0.63, 95% CI 0.43, 0.92; ages 50–64, aOR 0.25, 95% CI 0.16, 0.39; and ages 65 + aOR 0.04, 95% CI 0.02, 0.08). Being never married was associated with higher odds of past-year heroin or IMF use compared to being married in NSDUH (aOR 2.78, 95% 1.29, 5.98), and lower odds in NMURx (aOR 0.69, 95% CI 0.52, 0.91).

### Association of past-year heroin or IMF use with substance use, MOUD, and hospitalization

Both NSDUH and NMURx demonstrated similar patterns in past-year use of stimulants, benzodiazepines, and MOUD, showing higher odds of past-year heroin or IMF use (Table 3). Not using cigarettes was associated with a lower odds of past year heroin or IMF use in both surveys. NSDUH found no significant association (aOR 0.94, 95% CI 0.41, 2.13) between hospitalization and heroin or IMF use, whereas NMURx showed higher odds (aOR 1.90, 95% CI 1.44, 2.51).

**Table 3:** Comparing associations of heroin or illicitly manufactured fentanyl (IMF) use with clinical characteristics between NSDUH and NMUX in 2022. *Represents past year use. Stimulants were limited to cocaine and nonpharmaceutical amphetamines. Benzodiazepine use includes nonmedical use of prescription or nonpharmaceutical benzodiazepines

	NSDUH Adjusted Odds Ratio (95% CI)	NMURx Adjusted Odds Ratio (95% CI)
Cigarette Use (Ref.: Every day)		
Some days	0.94 (0.45, 1.96)	1.19 (0.94, 1.51)
Not at all / Blank	0.19 (0.08, 0.44)	0.46 (0.36, 0.58)
Cannabis Use (Ref: None)*	1 (0.53, 1.91)	1.19 (0.93, 1.51)
Heavy alcohol use (Ref: No)	1.08 (0.45, 2.6)	1.17 (0.8, 1.71)
Stimulant use (Ref: None)*	11.06 (6.22, 19.65)	27.52 (21.14, 35.83)
Benzodiazepine use (Ref: None)*	6.17 (3.68, 10.36)	4.1 (3.09, 5.44)
Medication for OUD use (Ref: None)*	22.06 (13.05, 37.3)	9.43 (6.31, 14.07)
Hospitalization in past year (Ref: None)	1.02 (0.47, 2.19)	1.89 (1.43, 2.49)

## Discussion

We demonstrate that past year heroin or IMF use was twice as prevalent in the NMURx (1.05%) survey compared to NSDUH (0.52%) survey, which amounts to an additional 1.3 million adults ages 18 + who used heroin or IMF in the past year. NMURx and NSDUH have different methodologies that potentially influence prevalence estimates of heroin or IMF use. First, considering the population characteristics, NMURx had a lower proportion of respondents reporting paid work in the last week and a higher proportion identifying as non-Hispanic White. Both of these factors are associated with higher levels of heroin use in previous waves of the NSDUH [21] and could account for the higher prevalence of heroin or IMF use in NMURx. NSDUH is a household-based survey whereas NMURx respondents could include people who are unhoused or living in a setting other than a house (e.g., residential treatment center or other group living) and have higher rates of substance use [22] which result in higher rates of heroin or IMF use in NMURx. Also, selection bias is potentially introduced through the opt-in design of the NMURx. Specifically, NMURx respondents had higher levels of education which could influence drug-related health literacy and awareness of IMF in the opioid supply or contamination of non-opioid substances with IMF. These characteristics potentially explain the higher prevalence of heroin or IMF use in NMURx relative to NSDUH.

Second, the question order and mode of data collection could influence respondents being more likely to disclose heroin or IMF use in NMURx relative to NSDUH. NMURx randomizes the order of questions to reduce differential bias in responses whereas NSDUH asks the questions in the same order questions. NSDUH IMF use questions are near the end of the survey, which could induce underreporting for IMF as respondents learn that answering in the affirmative to using a substance results in more questions [23]. NMURx respondents may be more comfortable reporting substance use since the survey is completely online and administered by a commercial survey firm as opposed to the NSDUH where a majority of respondents are completing the survey in the presence of an employee that is contracted by a federal government agency. Despite the NSDUH’s clear commitment to respondents confidentiality, it is possible that the distrust of the government and the increasing number of states with fentanyl-related laws impact a respondent’s willingness to disclose heroin or IMF use in a government survey [24]. 

Improving the accuracy and responsiveness to change in national substance use surveillance will require a complimentary approach, integrating multiple data collection systems. These may include national surveys, such as NSDUH and NMURx, toxicology testing results, and death certificate data. In addition, novel methods of collecting information such as wastewater sampling, using capture, recapture methods, or applying corrections to epidemiologic data potential methods to improve national estimates [25–27]. Small changes to existing systems, such as randomizing question blocks in the NSDUH or considering a grouped question design to reduce motivated underreporting [28], are now feasible since respondents are completing the questionnaire on a home device or in-person using computer adaptive questions. Iterative cognitive testing of new questions in NMURx and NSDUH would also support the content validity and reliability of responses. By improving existing data collection systems, expanding the range of data sources, and comparing results across systems, it is possible to provide more reliable population-level data on substance use.

When comparing patterns of associations between past-year heroin or IMF use and demographic and substance covariates across NMURx and NSDUH, there was generally agreement in the direction of association for most covariates. An exception was age where NMURx showed that older respondents were less likely to report past-year heroin or IMF, which was not replicated in NSDUH. We hypothesize that younger respondents might have been more likely to disclose heroin or IMF use in NMURx compared to NSDUH or be more sensitive to the order of the questions for the reasons described above. Stimulant (cocaine and methamphetamine) use and benzodiazepine use were strongly associated with past-year heroin or IMF use in both surveys, which increase the risk of overdose, highlighting the need for research on preventing, treating, and retaining people who use multiple substances in treatment [29]. The difference in the association of past-year heroin or IMF use and marital status between the surveys could be attributed to differences in social support systems accessible to respondents in each survey, which is a protective factor against substance use [30] and not directly assessed in the surveys.

The relative strengths and limitations of NSDUH and NMURx described above highlight the importance of integrating and triangulating data across multiple sources to improve population-level estimates of heroin or IMF use. For example, wastewater analysis generated estimates of opioid consumption (mg/day/1000 persons) and accurately predicted local overdose mortality [25]. Statistical methods, such as the Benchmark Multiplied Method, can also correct for underreporting and was applied to NSDUH and estimated the OUD prevalence was 5 times higher than reported in 2019 [26]. The drug supply is rapidly changing and national behavioral surveys will continue to provide key information about the most at-risk individuals but need to be able to rapidly adapt questions to represent the current drug supply.

Our study should be interpreted in light of limitations of making direct comparisons between the NSDUH and NMURx. While we sought to harmonize NSDUH and NMURx items, there were differences in the wording of items possibly interpreted differently by respondents. Bootstrapping provided a practical solution for managing differences between the survey designs and we acknowledge that it does not fully harmonize the methodologies and potentially introduces variability which lowers the precision of our estimates. The surveys were both fielded during 2022, yet the exact timing differed and there are possible seasonal behavioral patterns, societal, and economic events occurring around the time of collection which could impact estimates. In addition, differences in how survey data are processed, quality-controlled, and weighted can introduce variability in the results. Finally, while each survey is designed to represent the US population, the generalizability of the estimates is limited by their sampling frames, recruitment methodologies, and design choices for measuring the drug use constructs.

## Conclusions

The US is experiencing an overdose epidemic attributed to high-potency synthetic opioids like IMF. NSDUH included IMF use for the first time in 2022, with estimates of IMF use that are likely underestimates of the true prevalence of heroin or IMF use in the US. The NMURx suggested the prevalence of past year heroin or IMF use is twice as large as NSDUH estimates. Our findings suggest that no single nationally representative behavior survey is superior, rather integrating results from multiple national surveys and additional data sources is imperative to provide accurate and data about current substance use.

## Electronic supplementary material

Below is the link to the electronic supplementary material.

Supplementary Material 1

Supplementary Material 2

## Data Availability

No datasets were generated or analysed during the current study.
