# Supplementary material for: Comparison of heroin and fentanyl use in US nationally representative surveys
Source: Addict Sci Clin Pract. 2025 Feb 11;20:13. doi: 10.1186/s13722-025-00539-0 (PMC11812259; doi:10.1186/s13722-025-00539-0)

Supplement to “Comparison of Heroin and Fentanyl Use in US Nationally Representative Surveys.”

Supplement eTable 1: Harmonization of variables and response options from the National Survey on Drug Use and Health (NSDUH) and the Survey of Nonmedical Use of Prescription Drugs (NMURx)

| Topic | NSDUH | NMURx | Harmonized response |
| --- | --- | --- | --- |
| Age | (AGE3)  Categorical variable 1 = Respondent is 12 or 13 years old  2 = Respondent is 14 or 15 years old 3 = Respondent is 16 or 17 years old  4 = Respondent is between 18 and 20 years old  5 = Respondent is between 21 and 23 years old 6 = Respondent is 24 or 25 years old  7 = Respondent is between 26 and 29 years old  8 = Respondent is between 30 and 34 years old  9 = Respondent is between 35 and 49 years old  10 = Respondent is between 50 and 64 years old  11 = Respondent is 65 years old or older | Continuous variable in years | Recorded to Age groups:  18-25  26-34  35-49  50-64  65+ |
| Sex | (IRSEX)  1 = Male  2 = Female | Sex assigned at birth  Male  Female | Maintain Responses:  Male  Female |
| Income | (INCOME )  TOTAL FAMILY INCOME RECODE  1 = Less than $20,000  2 = $20,000 - $49,999  3 = $50,000 - $74,999  4 = $75,000 or More" | "What was your combined household income during the last 12 months? Select one  1= Less than $25,000  2= Between $25,000 and $49,999  3= Between $50,000 and $74,999  4 = Between $75,000 and $99,999  5 = $100,000 or more" | Collapsed NSDUH and NMURx to three categories:  -Less than $50,000  -$50,000 - $74,999  -$75,000 or More |
| Education | (EDUHIGHCAT)  1 = Less high school  2 = High school grad  3 = Some coll/Assoc Dg  4 = College graduate | 1 = Less than a high school diploma  2= Regular high school diploma, GED, or alternative credential  3 = Some college credit, but no degree  4 = Trade school  5 = Associate’s degree (for example, AA, AS)  6 =Bachelor’s degree (for example, BA, BS)  7 =Master’s degree (for example, MA, MS, MEng, Med, MSW, MBA  8 = Doctorate or Professional degree (for example, PhD, EdD, JD, MD | Recode NMURx to match NSDUH highest level of education.  -Less high school  -High school/GED  -Some college/assoc degree/trade  -College graduate |
| Married | (IRMARIT)  1 = Married  2 = Widowed  3 = Divorced or Separated  4 = Never Been Married  99 = LEGITIMATE SKIP Respondent is <= 14 years old | "1= Now Married  2 = Widowed  3 = Divorced  4 = Separated  5= Never Married" | Recode NMURx to match NSDUH marital categories:  -Married  -Widowed  -Divorced or Separated  -Never Been Married |
| Past week work | (WRKSTATWK2)  "WORK SITUATION IN PAST WEEK”  1 = Worked at full-time job, past week  2 = Worked at part time job, past week  3 = Has job or volunteer worker, did not work past wk  4 = Unemployed/on layoff, looking for work  5 = Disabled  6 = Keeping house full-time  7 = In school/training  8 = Retired  9 = Does not have a job, some other reason  98 = BLANK (NO ANSWER)  99 = LEGITIMATE SKIP " | "In the last week, did you work for pay at a job (or business)? Please answer “Yes” if you were temporarily absent for any reason. Select one.  1 = yes  0 = no" | Recode NSDUH to match NMURx reporting paid work in last week:  -Yes  -No |
| Cannabis | (IRMJRC)  MARIJUANA RECENCY - IMPUTATION REVISED  1 = Within the past 30 days  2 = More than 30 days ago but within the past 12 mos  3 = More than 12 months ago  9 = NEVER USED MARIJUANA" | Cannabis use in the last 12 months 1= yes 0 = no | Recode NSDUH to match NMURx to capture cannabis in the last year  -Yes  -No |
| Cigarette | (CIG30USE)  HOW MANY DAYS SMOKED CIG IN PAST 30 DAYS:  RANGE = 1 - 30  91 = NEVER USED CIGARETTES  93 = DID NOT USE CIGARETTES IN THE PAST 30 DAYS  94 = DON'T KNOW  97 = REFUSED  98 = BLANK (NO ANSWER)  (IRCIGRC)  TIME SINCE LAST SMOKED CIGARETTES  1 = Within the past 30 days  2 = More than 30 days ago but within the past 12 mos  3 = More than 12 months ago but within the past 3 yrs  4 = More than 3 years ago  9 = NEVER SMOKED CIGARETTES | "Do you NOW smoke cigarettes every day, some days, or not at all? Please answer ""Not at all"" if you have smoked fewer than 100 cigarettes in your life. Select one.  1= Every day  2= Some days  3= Not at all  4 = Don’t know" | Recode NSDUH to match NMURx cigarette use:  -Every day  -Some days  -Not at all  -Don’t know/Blank |
| Alcohol | (ALCUS30D)  "RC-NUMBER OF ALC BEVS DRANK PER USER IN PAST MONTH  RANGE = 1 - 90  975 = AT LEAST 4 OR 5 Logically assigned  985 = BAD DATA Logically assigned  991 = NEVER USED ALCOHOL  993 = DID NOT USE ALCOHOL IN THE PAST 30 DAYS  994 = DON'T KNOW  997 = REFUSED  998 = BLANK (NO ANSWER" | "How many standard drinks of alcohol do you drink per week? A standard drink is 12 oz. of beer, 5 oz. of wine, or 1.5 oz. of spirits or liquor. Select one.  1 = 0  2 = 1-7  3 = 8-14  4 = 15-21  5 = 22 or more" | Use NSDUH and NMURx to create binary variables for heavy alcohol use based on the NIAAA sex-specific number of drinks defining heavy alcohol use:  (≥ 15 drinks per week for men; ≥ 8 drinks per week for women)  -Yes  -No |
| Stimulant | (IRMETHAMREC)  "METHAMPHETAMINE RECENCY - IMPUTATION REVISED  1 = Within the past 30 days  2 = More than 30 days ago but within the past 12 mos  3 = More than 12 months ago  9 = NEVER USED METHAMPHETAMINE "  (IRCOCRC )  "TIME SINCE LAST USED COCAINE  1 = Within the past 30 days  2 = More than 30 days ago but within the past 12 mos  3 = More than 12 months ago  9 = NEVER USED COCAINE "  (IRCRKRC)  "CRACK RECENCY - IMPUTATION REVISED  1 = Within the past 30 days  2 = More than 30 days ago but within the past 12 mos  3 = More than 12 months ago  9 = NEVER USED COCAINE " | Illicit stimulant use in the last 12 months.  Derived from report of most recent time used was either “within the last week”, “1 to 4 weeks ago”, “1 to 3 months ago”, “4 to 12 months ago”  Survey options included: “Amphetamine not made by a drug company”, “Cocaine Powder”, “Crack Cocaine”, or “Methamphetamine  1= Yes  2= No | Recode NSDUH to match NMURx to note any past year use of illicit stimulants (cocaine and methamphetamines)  -Yes  -No |
| Benzodiazepine | (IRSEDNMREC)  "SEDATIVE MISUSE RECENCY - IMPUTATION REVISED  1 = Within the past 30 days  2 = More than 30 days ago but within the past 12 mos  3 = More than 12 months ago  9 = NEVER MISUSED SEDATIVES"  (IRTRQNMREC)  "TRANQUILIZER MISUSE RECENCY - IMPUTATION REVISED  1 = Within the past 30 days  2 = More than 30 days ago but within the past 12 mos  3 = More than 12 months ago  9 = NEVER MISUSED TRANQUILIZERS" | Derived: Any use of a prescription benzodiazepine in the last 12 months – medical or non-medical use  Included prescription benzodiazepines: alprazolam, chlordiazepoxide, clobazam, clonazepam, clorazepate, daridorexant, diazepam, estazolam, flurazepam, lemborexant, lorazepam, midazolam, oxazepam, quazepam, remimazolam, sodium oxybate, suvorexant, temazepam, and triazolam.  1=Yes  2= No | Recode NSDUH to match NMURx noting any past year use of nonprescribed benzodiazepines.  -Yes  -No |
| Self-reported health status | (HEALTH2)  . = Unknown (Otherwise)  1 = Excellent (HEALTH=1)  2 = Very Good (HEALTH=2)  3 = Good (HEALTH=3)  4 = Fair/Poor (HEALTH=4,5)" | Would you say your health in general is excellent, very good, good, fair, or poor? Drag the slider to answer the question 1= Poor 2= Fair 3 = Good 4 = Very good 5 = Excellent | Recode NMURx to match NSDUH  -Fair/Poor  -Good  -Very good  -Excellent |
| Medication for Opioid Use Disorder | (IRSUTRXDRG)  "USED RX MED TO HELP CUT BACK/STOP HER/PNR USE - IMP REV  0 = No  1 = Yes" | In the last 12 months, have you been prescribed any medications for opioid dependence? Please check all that apply.  / Yes, buprenorphine  / Yes, methadone  1= yes  0 =no" | Recode NMURx to capture any MOUD and match NSDUH  -Yes  -No |
| Hospitalization | (NMNGTHS2)  "In the past year, how NIGHTS STAYED IN HOSPITAL OVERNIGHT OR LONGER  RANGE = 1 - 30  31 = 31 or more.  985 = BAD DATA Logically assigned  994 = DON'T KNOW  997 = REFUSED  998 = BLANK (NO ANSWER  999 = LEGITIMATE SKIP" | During the last 12 months, were you a patient in a hospital overnight? Select one. 1= yes 0 = no | Recode NSDUH to match NMURx indicating any overnight hospitalization in the past year.  -Yes  -No |

eTable 2: Comparison of the Population Characteristics and Substance Use between the 2022 National Survey of Drug Use and Health (NSDUH) and 2022 Survey of Non-Medical Use of Prescription Drugs (NMURx), ages 18+.

|  | NSDUH  n = 47,100  Weighted n = 256,281,676 | | NMURx  n = 59,041  Weighted n = 259,008,595 | |  |
| --- | --- | --- | --- | --- | --- |
|  | Frequency | Weighted Percentage, % (95% CI) | Frequency | Weighted Percentage, % (95% CI) | Weighted Proportion Absolute Difference  (corrected 95% CI*) |

| Age group |  |  |  |  |  |
| --- | --- | --- | --- | --- | --- |
| 18-25 | 14307 | 13.57 (13.03, 14.13) | 4062 | 12.96 (12.52, 13.41) | -0.62 (-1.21, 0.01) |
| 26-34 | 9645 | 15.69 (14.97, 16.43) | 7513 | 16.30 (15.89, 16.72) | 0.61 (-0.02, 1.25) |
| 35-49 | 12588 | 24.42 (23.75, 25.1) | 17565 | 24.20 (23.80, 24.6) | -0.21 (-1.00, 0.56) |
| 50-64 | 5369 | 24.16 (23.42, 24.92) | 16882 | 24.87 (24.48, 25.27) | 0.72 (-0.22, 1.65) |
| 65+ | 5191 | 22.15 (21.23, 23.1) | 13019 | 21.67 (21.27, 22.07) | -0.49 (-1.35, 0.42) |
| Sex |  |  |  |  |  |
| Male | 20852 | 48.81 (47.93, 49.69) | 27596 | 48.82 (48.31, 49.33) | -0.01 (-1.00,1.00) |
| Female | 26248 | 51.19 (50.31, 52.07) | 31445 | 51.18 (50.67, 51.69) | 0.01 (-1.00, 1.00) |
| Race/Ethnicity |  |  |  |  |  |
| Non-Hispanic White | 28426 | 61.72 (60.17, 63.25) | 43385 | 70.14 (69.65, 70.62) | **8.43 (7.45, 9.40)** |
| Non-Hispanic Black | 5421 | 12.07 (11.28, 12.91) | 5550 | 10.44 (10.11, 10.78) | **-1.63 (-2.25, -1.02)** |
| Non-Hispanic Native Am | 629 | 0.49 (0.40, 0.60) | 933 | 1.37 (1.26, 1.49) | **0.88 (0.73, 1.02)** |
| Non-Hispanic Native HI | 204 | 0.45 (0.32, 0.63) | 185 | 0.36 (0.3, 0.44) | -0.08 (-0.24, 0.05) |
| Non-Hispanic Asian | 2521 | 6.07 (5.49, 6.72) | 2126 | 5.15 (4.91, 5.41) | **-0.93 (-1.43, -0.43)** |
| Another Non-Hispanic Race or Multiple Race/Ethnicities | 1756 | 1.92 (1.77, 2.09) | 569 | 0.99 (0.89, 1.10) | **-0.93 (-1.15, -0.73)** |
| Hispanic | 8143 | 17.27 (16.04, 18.57) | 6293 | 11.55 (11.20, 11.90) | **-5.73 (-6.47, -4.97)** |
| Income |  |  |  |  |  |
| Less than $50,000 | 20817 | 41.87 (40.73, 43.02) | 27697 | 44.66 (44.16, 45.17) | **4.75 (3.98, 5.49)** |
| $50,000 - $74,999 | 6895 | 15.31 (14.69, 15.95) | 11071 | 20.06 (19.65, 20.47) | **2.79 (1.78, 3.77)** |
| $75,000 or More | 19388 | 42.82 (41.69, 43.95) | 20273 | 35.28 (34.80, 35.77) | **-7.54 (-8.52, -6.57)** |
| Education |  |  |  |  |  |
| Less high school | 4775 | 9.36 (8.91, 9.83) | 2096 | 3.00 (2.82, 3.19) | -6.36 (-6.93, -5.8) |
| High school/general education diploma | 12065 | 27.13 (26.24, 28.04) | 12476 | 20.20 (19.79, 20.62) | **-6.93 (-7.79, -6.03)** |
| Some college/associates degree/trade | 13964 | 33.23 (32.29, 34.18) | 21259 | 35.47 (34.98, 35.95) | **5.17 (4.27, 6.07)** |
| College graduate (bachelors or higher) | 16296 | 33.2 (32.3, 34.2) | 23210 | 41.34 (40.84, 41.84) | **8.12 (7.21, 9.09)** |
| Marital Status |  |  |  |  |  |
| Married | 19520 | 48.71 (47.77, 49.66) | 29854 | 48.68 (48.17, 49.19) | -0.03 (-1.09, 0.94) |
| Widowed | 1340 | 5.67 (5.13, 6.25) | 3927 | 5.67 (5.46, 5.89) | -0.01 (-0.51, 0.49) |
| Divorced or Separated | 4673 | 14.06 (13.43, 14.71) | 10116 | 13.98 (13.66, 14.31) | -0.08 (-0.83, 0.63) |
| Never Been Married | 21567 | 31.56 (30.58, 32.56) | 15144 | 31.68 (31.17, 32.19) | 0.11 (-0.8, 1.03) |
| Work in past week (Yes) | 27068 | 52.78 (51.93, 53.63) | 23335 | 43.51 (43, 44.01) | **-9.28 (-10.32, -8.31)** |

| Private health insurance (Yes) | 28543 | 60.72 (59.92, 61.52) | 34814 | 61.3 (60.8, 61.8) | -0.57 (-1.56, 0.37) |
| --- | --- | --- | --- | --- | --- |

| Heroin use, past year | 192 | 0.39 (0.30, 0.52) | 635 | 0.60 (0.54, 0.67) | **0.21 (0.09, 0.32)** |
| --- | --- | --- | --- | --- | --- |
| IMF use, past year | 120 | 0.24 (0.18, 0.33) | 801 | 0.74 (0.67, 0.81) | **0.49 (0.39, 0.60)** |
| Heroin or IMF use, past year | 250 | 0.52 (0.40, 0.69) | 1120 | 1.05 (0.97, 1.14) | **0.53 (0.38, 0.67)** |
| Cigarette Use |  |  |  |  |  |
| Every day | 3767 | 9.28 (8.83, 9.75) | 13531 | 9.11 (8.93, 9.29) | -0.16 (-0.74, 0.35) |
| Some days | 3222 | 6.54 (6.21, 6.88) | 6186 | 2.89 (2.81, 2.98) | **-3.64 (-4.04, -3.23)** |
| Not at all | 40064 | 84.07 (83.47, 84.65) | 37211 | 87.9 (87.69, 88.11) | **3.82 (3.19, 4.49)** |
| Don't know/Blank | 47 | 0.11 (0.06, 0.20) | 2113 | 0.09 (0.09, 0.1) | -0.02 (-0.1, 0.04) |
| Cannabis use (Yes) | 12796 | 22.95 (22.18, 23.74) | 14705 | 21.38 (20.97, 21.81) | **-1.58 (-2.38, -0.79)** |
| Heavy Alcohol use (Yes) | 4675 | 8.11 (7.61, 8.63) | 3747 | 5.14 (4.94, 5.35) | **-2.97 (-3.45, -2.47)** |
| Nonmedical Benzodiazepine use (Yes) | 778 | 1.39 (1.21, 1.59) | 1944 | 2.09 (1.97, 2.23) | **0.7 (0.49, 0.91)** |
| Nonmedical Stimulant use (Yes) | 1479 | 2.86 (2.49, 3.27) | 2982 | 3.02 (2.87, 3.17) | 0.16 (-0.16, 0.48) |
| Medications for OUD (Yes) | 466 | 0.96 (0.81, 1.14) | 712 | 0.56 (0.51, 0.62) | **-0.40 (-0.57, -0.24)** |
| Self-reported health status |  |  |  |  |  |
| Fair/Poor | 5821 | 15.17 (14.39, 15.98) | 11468 | 15.04 (14.70, 15.38) | -0.13 (-0.86, 0.60) |
| Good | 14732 | 32.27 (31.37, 33.19) | 23027 | 39.64 (39.14, 40.14) | 7.35 (6.36, 8.35) |
| Very good | 17543 | 35.35 (34.43, 36.29) | 18136 | 34.21 (33.72, 34.70) | -1.15 (-2.09, -0.23) |
| Excellent | 9004 | 17.21 (16.46, 17.98) | 6410 | 11.12 (10.8, 11.46) | -6.07 (-6.75, -5.39) |
| Hospitalization (yes) | 3368 | 8.20 (7.68, 8.76) | 6843 | 9.48 (9.20, 9.77) | **1.28 (0.71, 1.85)** |
| Bolded values show statistically significant absolute differences. Abbreviation: opioid use disorder (OUD)  *Bonferroni corrected confidence interval. Setting significant level of α = 0.05 and accounting for 43 comparisons, the corrected significant level (α) is equal to 0.05/42 = 0.0011 and we constructed 99.88% confidence intervals. The absolute difference formula: NMURx prevalence minus NSDUH prevalence. | | | | | |

eFigure 1: Average Marginal Effects of demographic and socioeconomic factors with the reporting past year use of either heroin or illicitly manufactured fentanyl from the 2022 NSDUH and 2022 NMURx.


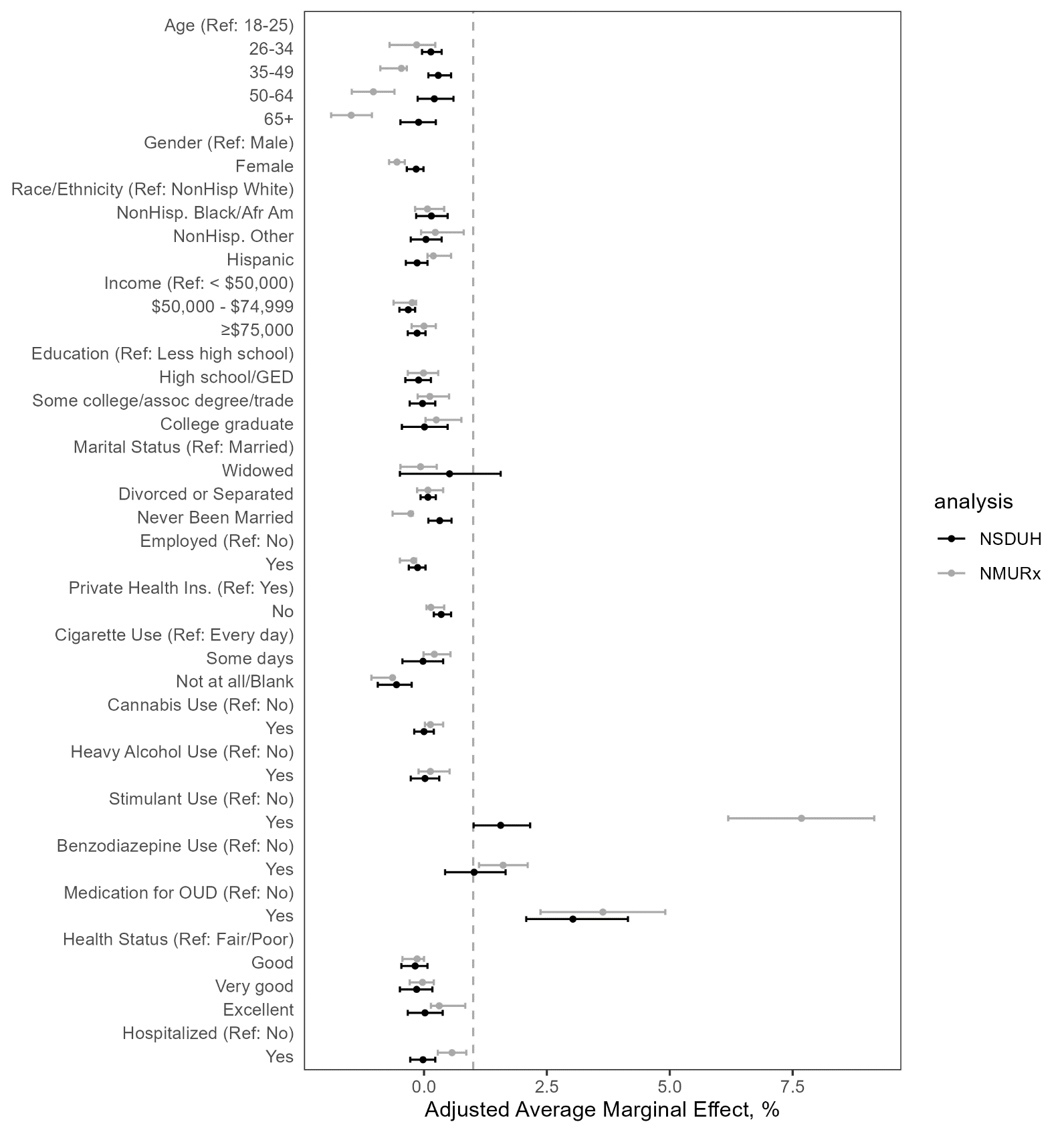

Supplement: Supplementary file 2 — Supplementary Material 2 [file 13722_2025_539_MOESM2_ESM.docx]
